# Genomic Analyses of Acute Flaccid Myelitis Cases among a Cluster in Arizona Provide Further Evidence of Enterovirus D68 Role

**DOI:** 10.1128/mBio.02262-18

**Published:** 2019-01-22

**Authors:** Jolene R. Bowers, Michael Valentine, Veronica Harrison, Viacheslav Y. Fofanov, John Gillece, Josie Delisle, Bethany Patton, James Schupp, Krystal Sheridan, Darrin Lemmer, Scott Ostdiek, Harlori K. Bains, Jennifer Heim, Tammy Sylvester, Siru Prasai, Melissa Kretschmer, Nicole Fowle, Kenneth Komatsu, Shane Brady, Susan Robinson, Kathryn Fitzpatrick, Gholamabbas Amin Ostovar, Eric Alsop, Elizabeth Hutchins, Kendall Jensen, Paul Keim, David M. Engelthaler

**Affiliations:** a Pathogen and Microbiome Division, Translational Genomics Research Institute, Flagstaff, Arizona, USA; b School of Informatics Computing and Cyber Systems, Northern Arizona University, Flagstaff, Arizona, USA; c Phoenix Children’s Hospital, Phoenix, Arizona, USA; d Maricopa County Department of Public Health, Phoenix, Arizona, USA; e Arizona Department of Health Services, Phoenix, Arizona, USA; f Maricopa Integrated Health System, Phoenix, Arizona, USA; g Neurogenomics Division, Translational Genomics Research Institute, Phoenix, Arizona, USA; h Pathogen and Microbiome Institute, Northern Arizona University, Flagstaff, Arizona, USA; Icahn School of Medicine at Mount Sinai

**Keywords:** AFM, EV-D68, acute flaccid myelitis, enterovirus, genomics, metagenomics

## Abstract

Enteroviruses are a common cause of respiratory and gastrointestinal illness, and multiple subtypes, including poliovirus, can cause neurologic disease. In recent years, enterovirus D68 (EV-D68) has been associated with serious neurologic illnesses, including acute flaccid myelitis (AFM), frequently preceded by respiratory disease. A cluster of 11 suspect cases of pediatric AFM was identified in September 2016 in Phoenix, AZ. To determine if these cases were associated with EV-D68, we performed multiple genomic analyses of nasopharyngeal (NP) swabs and cerebrospinal fluid (CSF) material from the patients, including real-time PCR and amplicon sequencing targeting the EV-D68 VP1 gene and unbiased microbiome and metagenomic sequencing. Four of the 11 patients were classified as confirmed cases of AFM, and an additional case was classified as probable AFM. Real-time PCR and amplicon sequencing detected EV-D68 virus RNA in the three AFM patients from which NP swabs were collected, as well as in a fourth patient diagnosed with acute disseminated encephalomyelitis, a disease that commonly follows bacterial or viral infections, including enterovirus. No other obvious etiological causes for AFM were identified by 16S or RNA and DNA metagenomic sequencing in these cases, strengthening the likelihood that EV-D68 is an etiological factor. Herpes simplex viral DNA was detected in the CSF of the fourth case of AFM and in one additional suspect case from the cluster. Multiple genomic techniques, such as those described here, can be used to diagnose patients with suspected EV-D68 respiratory illness, to aid in AFM diagnosis, and for future EV-D68 surveillance and epidemiology.

## Abstract (precis)

Enteroviruses frequently result in respiratory and gastrointestinal illness; however, multiple subtypes, including poliovirus, can cause severe neurologic disease. Recent biennial increases (i.e., 2014, 2016, and 2018) in cases of non-polio acute flaccid paralysis have led to speculations that other enteroviruses, specifically enterovirus D68 (EV-D68), are emerging to fill the niche that was left from poliovirus eradication. A cluster of 11 suspect cases of pediatric acute flaccid myelitis (AFM) was identified in 2016 in Phoenix, AZ. Multiple genomic analyses identified the presence of EV-D68 in the majority of clinical AFM cases. Beyond limited detection of herpesvirus, no other likely etiologies were found in the cluster. These findings strengthen the likelihood that EV-D68 is a cause of AFM and show that the rapid molecular assays developed for this study are useful for investigations of AFM and EV-D68.

## INTRODUCTION

Enteroviruses comprise a broad assortment of viruses, causing a diverse array of disease manifestations involving respiratory, skin, neurologic, and gastrointestinal sites, but infections are also frequently asymptomatic (1). Before poliovirus (enterovirus C) vaccine implementation, upwards of 600,000 children worldwide were paralyzed each year by poliovirus (2). The recent increase in cases of non-polio acute flaccid paralysis (AFP) has led to speculations that other enteroviruses are emerging to fill the niche vacated via eradication of poliovirus (3, 4). Previously, enterovirus A71 (EV-A71) and several enterovirus B serotypes, mainly echoviruses and coxsackieviruses, were implicated in hundreds of cases of AFP following the success of global polio vaccine campaigns (3).

Enterovirus D68 (EV-D68), first characterized in 1962, did not emerge as a major cause of respiratory infections until 2009 (5). Shortly thereafter, EV-D68 was correlated with clusters of an AFP termed acute flaccid myelitis (AFM), which presents with demonstrable lesions in the spinal cord or brain stem, distinguishing it from other forms of AFP (6). In 2014, EV-D68 was detected in many AFM patients (7–15) and AFM cases temporally corresponded to peaks in EV-D68 infections (11, 16), although the upsurge in EV-D68 was orders of magnitude larger than the increase in AFM.

In 2016, 149 people in 39 U.S. states were classified as confirmed cases of AFM (17), correlating to regional outbreaks that were part of another nationwide seasonal increase in EV-D68 infection (15, 18, 19): this is likely a low estimate, and the number could rise if additional suspect cases from 2016 are reviewed (20). Late in the summer of 2016, physicians in Phoenix, AZ, noted a cluster of children with symptoms consistent with AFM (20). We employed highly sensitive analyses on clinical samples from that outbreak to identify possible etiologic agents, including targeted PCR and amplicon sequencing for EV-D68 and 16S microbiomic analysis and metagenomic analyses for other potential causes of AFM or other neurologic disease.

## RESULTS

### Clinical results.

Table 1 includes clinical information for the 11 suspect pediatric AFM cases. Four of the eleven children met the case definition of confirmed AFM after medical record reviews and in-person interviews. One child met the case definition of probable AFM after medical record review showed pleocytosis without an alternative diagnosis despite a normal magnetic resonance imaging (MRI). Three of these five children had asthma, and another had a family history of asthma (20). The other children’s diagnoses recorded here were the leading differential diagnoses at the time data were abstracted and included acute disseminated encephalomyelitis (ADEM), multiple sclerosis (MS), Guillain-Barré syndrome (GBS), and neuromyelitis optica (NMO) (Table 1). Patient 4, diagnosed with confirmed AFM, was positive for coxsackievirus A10 in a stool sample collected 28 days post-onset of focal limb weakness. Genomic analyses were blinded from these results to prevent testing and analysis bias.

**TABLE 1:** Patient clinical symptoms and initial testing results^*a*^

Patient no.	Age (yr)	Case classification or leading differential diagnosis	Preceding illness	No. of days of illness to limb weakness onset	CSF white blood cells/mm^3^	No. of limbs affected	MRI result(s)
1	3.5	Confirmed AFM	Respiratory, fever	2	50	1, LUE	T2 signal abnormalities in anterior and posterior columns of the central gray cervical cord
2	10	Confirmed AFM	Respiratory	5	150	4	T2 signal abnormality with anterior and posterior involvement, contiguous through multiple levels of the cord
3	4	Confirmed AFM	Respiratory, fever	2	207	3, BUE, RLE	T2 signal abnormality in the anterior horn of the central gray cord
4	9	Confirmed AFM	GI, fever	2	115	4	Anterior horn signal abnormality extending four cervical levels
5	12	Probable AFM	Respiratory	10	7	1, LUE	Normal
6	12	Unknown	None	NA	5	2, BLE	T3–T7, T11–T12 gray matter affected
7	7.5	ADEM	Respiratory, fever	0	7	1, RUE	C4, C6, T11 gray/white matter affected
8	17	NMO	Respiratory	10	22	2, BLE	C3–C7, T1, T10–T11 gray/white matter affected
9	6.5	GBS	None	NA	1	2, BLE	Normal
10	14	MS/ADEM	GI	6	5	2, BLE	C1–C5, C7, T1, T4–T6, T11–T12 gray/white matter affected
11	1.5	Unknown	GI, fever	0	0	2, BLE	T4–T7 gray/white matter affected; edema of conus

a Diagnoses reflect whether the AFM case definition was met (confirmed AFM or probable AFM) or not, in which case the leading differential diagnosis at the time of data abstraction was recorded. MRI results for the patients who met the AFM case definitions are those described by the CDC neurology subject matter expert, while MRI results for patients who did not meet the AFM case definitions are those described by the attending radiologist. Other specimen testing included PCR for herpes simplex virus, West Nile virus, human herpesvirus 6, Epstein Barr virus, and Mycoplasma pneumoniae, as well as serology for West Nile virus and the VDRL test for syphilis (20). AFM, acute flaccid myelitis; ADEM, acute disseminated encephalomyelitis; NMO, neuromyelitis optica; GBS, Guillain-Barré syndrome; MS, multiple sclerosis; LUE, left upper extremity; BUE, bilateral upper extremity; RLE, right lower extremity; BLE, bilateral lower extremity; RUE, right upper extremity; NA, not applicable; GI, gastrointestinal.

### EV-D68 real-time PCR and targeted amplicon sequencing.

From the 11 suspect AFM patients, six had nasopharyngeal (NP) swabs available for genomic analysis. Four of these six tested positive for EV-D68 by both real-time PCR and amplicon sequencing (Table 2). Three of these were from patients subsequently classified as confirmed cases of AFM, and one was from a patient with differential diagnoses of ADEM or MS. No NP swab was available from patient 4, who was the fourth confirmed AFM patient and was stool positive for coxsackievirus A10 by the clinical laboratory. None of the CSF specimens tested positive for EV-D68, despite the fact that two AFM patients had CSF drawn 1 day following onset of their focal limb weakness (Table 2). Like patient 4 (confirmed AFM), no NP swab from the single probable AFM case (patient 5) was available for genomic analysis.

**TABLE 2:** Study specimen information and molecular results of RNA analysis data^*a*^

Patient no.	Diagnosis	Specimen ID	Specimen type	No. of days of limb weakness to specimen collection	EV-D68 Taqman *C_T_*	EV-D68 amplicon read count
1	AFM	48135	CSF	2	Neg	0
		48136	NP swab	4	32.1	8
2	AFM	48138	CSF	1	Neg	0
		48137	NP swab	1	29.8	16
3	AFM	48143	CSF	1	Neg	0
		48145	NP swab	7	26.2	242
4	AFM	48127	CSF	6	Neg	0
5	AFM^*b*^	48130	CSF	19	Neg	0
6	Unknown	48124	CSF	5	Neg	0
7	ADEM	48134	CSF	7	Neg	0
		48132	NP swab	8	Neg	0
8	NMO	48125	CSF	5	Neg	0
9	GBS	48131	CSF	32	Neg	0
10	MS/ADEM	48140	CSF	9	Neg	0
		48139	NP swab	8	31.0	36
11	Unknown	48148	CSF	15	Neg	0
		48147	NP swab	14	Neg	0

a The number of days from initial respiratory or gastrointestinal illness to specimen collection is the sum of the number of days of illness to limb weakness onset (Table 1) and the number of days of limb weakness to specimen collection. AFM, acute flaccid myelitis; ADEM, acute disseminated encephalomyelitis; NMO, neuromyelitis optica; GBS, Guillain-Barré syndrome; MS, multiple sclerosis; *C_T_*, threshold cycle; Neg, negative.

b Probable.

Though this sample set is too small to draw statistical conclusions from the data, sensitivity, specificity, and overall agreement of the PCR-based results on NP swabs when available, and on CSF otherwise, with the clinical diagnoses as the reference standard, were calculated. With *n* = 10, considering one patient was without a clinical diagnosis, values with the Wilson score-based 95% confidence intervals (CIs) are as follows: sensitivity, 0.80 (95% CI, 0.38 to 0.96); specificity, 1.0 (95% CI, 0.57 to 1.0); and overall agreement, 0.90 (95% CI, 0.60 to 0.98). The 95% CIs of these measures show that the EV-D68 assay results are consistent with AFM diagnosis, as the lower bounds do not include 0.

Polymorphisms were identified among the amplicon sequences of each sample. Not including the primer regions, the VP1 gene sequence of sample 48136 was one single nucleotide polymorphism (SNP) different from a 2015 Japan isolate (GenBank accession no. LC203572) and several 2013 to 2014 Philippines isolates (accession no. AB992437, AB992417, KX789257, and KX789240). Sample 48137 was one SNP different from the five above (at a different locus) and from a 2016 Denmark isolate (accession no. KY457569). Samples 48139 and 48145 were a perfect match to many global strains from 2013 to 2017. Interpretation of these data is limited, however, as we only sequenced a fragment of the VP1 gene (Table 3), and sequencing error may have impacted consensus sequences due to low coverage in three of four samples (Table 2).

**TABLE 3:** Primers and probes used in this study^*a*^

Assay component	Name	Sequence
Real-time PCR primer	EVD68_F1	CRTGGGTCTTCCTGACTTRAC
	EVD68_F2	AYRGGCCTTCCTGACTTGAC
	EVD68_F3	YGTGGGTCTTCCTGACTTGAC
	EVD68_R1	RCCTGAYTGCCARTGGAATG
	EVD68_R2	GCCTGAYTGCCARTGGAAYG
Real-time PCR probe	EVD68_FB1	6FAM-CARGCAATGTTTGTACCBACTGGTGC-BHQ
	EVD68_FB2	6FAM-CAAGCAATGTTYGTRCCCACTGGTGC-BHQ
Amplicon sequencing primer	EVD68-UT_F1	ACCCAACTGAATGGAGCCRTGGGTCTTCCTGACTTRAC
	EVD68-UT_F2	ACCCAACTGAATGGAGCAYRGGCCTTCCTGACTTGAC
	EVD68-UT_F3	ACCCAACTGAATGGAGCYGTGGGTCTTCCTGACTTGAC
	EVD68-UT_R1	ACGCACTTGACTTGTCTTCRCCTGAYTGCCARTGGAATG
	EVD68-UT_R2	ACGCACTTGACTTGTCTTCGCCTGAYTGCCARTGGAAYG
16S amplicon sequencing primer	UT1-S-D-Bact-0341-b-S-17^*b*^	ACCCAACTGAATGGAGCCCTACGGGNGGCWGCAG
	UT2-S-D-Bact-0785-a-A-21^*b*^	ACGCACTTGACTTGTCTTCGACTACHVGGGTATCTAATCC

a The EV-D68 assays target the VP1 gene, and the real-time PCR results in a 94-bp amplicon. Underlined sequences are the universal tails. 6FAM, 6-carboxyfluorescein; BHQ, black hole quencher.

b Primers without these universal tails are from reference 70.

### 16S microbiomic analysis.

**FIG 1:**
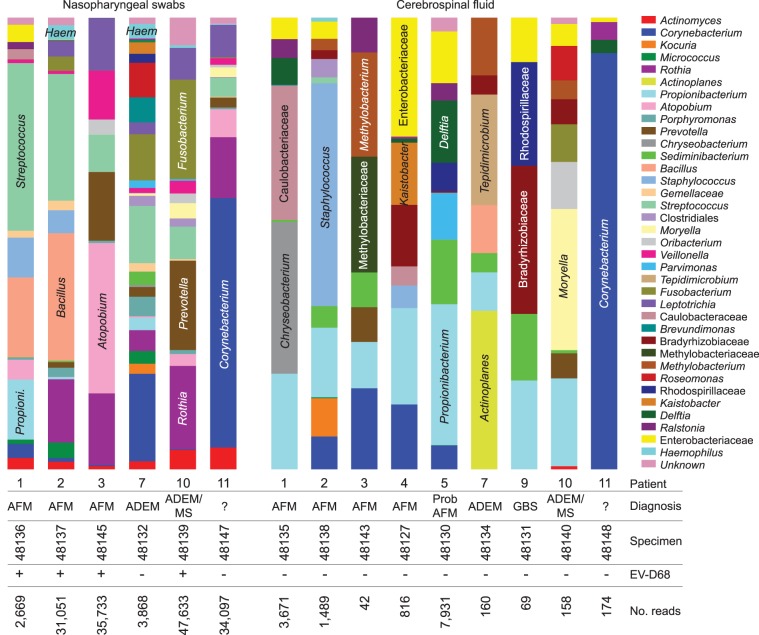
Results of microbiomic analysis by 16S rRNA partial gene sequencing after removing background taxa present in the 91 reads of a reagent-only negative-control sample, showing normal flora carriage in the NP swabs in all of the patients regardless of their neurologic disease diagnosis and probable contaminants in the CSF samples. The sample size is too small to detect differences in NP bacterial communities. ?, unknown.

The mean number of 16S sequence reads generated in the nine cerebrospinal fluid (CSF) samples analyzed for bacterial population was 1,612, with five samples having <200 reads indicating low bacterial loads (Fig. 1). Of the six NP swabs analyzed for bacterial population, two samples generated <4,000 reads, while the other four averaged 37,128 reads. The 16S PCR reagent blank (negative control) yielded 91 reads.

The bacterial composition of all NP swabs, considering all taxa at ≥1% of the total after the reagent blank taxa were removed, was characterized by normal upper respiratory flora, such as Corynebacterium, Bacillus, Propionibacterium, Streptococcus, Fusobacterium, Prevotella, Atopobium, Rothia, Veillonella, Leptotrichia, and Haemophilus (Fig. 1). Five of the six NP swabs contained a small number of unknown taxa. The variation in composition between patients appears analogous to the variation seen among healthy subjects (21, 22). Four of the six NP swabs were collected from patients with recent respiratory illness. However, the swabs were collected between 6 and 9 days after the onset of the respiratory illness. The other two patients (10 and 11) had gastrointestinal illness.

There was no evidence of CSF bacterial infection in any patients, as 16S read counts were low and no specimens were dominated by one organism, except sample 48148, characterized by *Corynebacterium*, a known CSF culture contaminant (23) and laboratory contaminant (24) (Fig. 1). The low read counts likely highlighted several contaminants introduced during sample processing, as documented previously (24). Bacterial taxa found in the CSF specimens included *Propionibacterium*, *Bacillus* and Enterobacteriaceae, all known to be CSF culture contaminants (23, 25, 26) and laboratory contaminants (24). Although Enterobacteriaceae is a family containing well-known pathogens, the number of 16S reads matching these taxa was significantly lower than would be expected in an active CSF infection. Also found were Chryseobacterium, Delftia, Methylobacterium, Ralstonia, Roseomonas, Caulobacteriaceae, and Bradyrhizobiaceae, all known laboratory contaminants (24), but also recently shown to be part of the skin microbiome, as are Staphylococcus, Prevotella, and Sediminibacterium (27, 28), and thus, could be specimen collection contaminants. Some organisms were present in both the NP bacterial population and in the CSF data of a patient, such as Moryella, *Fusobacterium*, and Oribacterium in patient 10, suggesting possible crossover of these organisms to the patient’s skin and contamination of the CSF specimen. Parvimonas, part of the normal oral flora, was found in one CSF sample, suggesting another possible transfer from the skin. Other organisms, generally not associated with clinical disease, were found in some CSF samples at higher proportion (Actinoplanes, Tepidimicrobium, Rhodospirillaceae, and Kaistobacter), but total read counts were still relatively low for those samples (<200 reads); thus, their presence indicates contamination. Two CSF samples had reads from unknown taxa.

No obvious or common etiology for neurologic disease or respiratory illness was found from the bacterial population analyses. There were no significant taxa from interpatient comparative analysis nor comparisons between EV-D68-positive and EV-D68-negative samples. Conclusive elimination of a bacterial role is limited, however, due to the small sample size and lack of healthy control data.

### DNA and RNA metagenomic analyses.

Total RNA libraries from 11 CSF and 6 NP samples and total DNA libraries from 2 CSF and 3 NP samples were sequenced (see Table S1 in the supplemental material). In both the CSF and NP swab RNA and DNA metagenomic data samples, human sequence reads were most common. Microorganism metagenomic results largely agreed with the 16S microbiomic analyses in organisms identified for the NP swabs and in identification of probable contamination in the CSF samples. From the metagenomic analyses, like the microbiomic analysis, no obvious bacterial etiology was found for the neurological illnesses in this outbreak.

10.1128/mBio.02262-18.2 TABLE S1 RNA and DNA metagenomic results from three analysis tools. For MTS, the top five hits at >0.1% of the total signature hits after removal of human reads are listed, for GOTTCHA, the top five hits of the bacterial and viral databases with >100 reads are listed, and for MetaPhlAn, the top five or all hits of the marker library at >1.0% of all hits are listed (minus hits to plant viruses, which were found in all samples by MetaPhlAn), except for taxa in parentheses, which are the top hits below the abundance threshold. Each row includes the samples from a single patient. Results were from RNA unless noted as DNA. Samples in which EV-D68 was detected by any method are colored blue, and CSF samples from patients diagnosed with AFM are colored red. In the MTS data, taxa in blue were also found in the 16S microbiomic data. In the GOTTCHA and MetaPhlAn data, species in purple were also found by MTS. Download Table S1, XLSX file, 0.1 MB. Copyright © 2019 Bowers et al. 2019 Bowers et al. This content is distributed under the terms of the Creative Commons Attribution 4.0 International license.

Three different sequence analysis tools were employed to characterize the organism composition in each metagenomic sample for robustness and comprehensiveness. Most significantly, herpes simplex virus (HSV), a known neuroinvasive pathogen, was the top viral hit (from the MTS tool analysis) in two of the CSF DNA metagenomic samples from patients 4 and 8, samples 48127 and 48125 (Table S1). Patient 8 was diagnosed with NMO, and patient 4, an AFM patient, was noted to have a cold sore at the time of examination. Neither of these patients had NP swabs available for testing. Detailed methods and results from these tools can be found in the supplemental material (see Text S1).

10.1128/mBio.02262-18.1 TEXT S1 Supplemental methods. A complete description of the metagenomic analysis of DNA and RNA from clinical samples is provided. Download Text S1, DOCX file, 0.1 MB. Copyright © 2019 Bowers et al. 2019 Bowers et al. This content is distributed under the terms of the Creative Commons Attribution 4.0 International license.

### Targeted query of metagenomic data.

**FIG 2:**
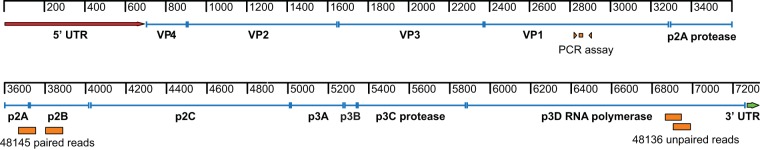
EV-D68 genome map with locations of the real-time PCR/amplicon sequencing assay and the metagenomic read alignments of two NP swab samples, 48136 and 48145, from children diagnosed with AFM. The unpaired reads from 48136 cover 132 bases of the p3D gene and overlap for 48 bases. The sequence was a perfect match to several 2016 genomes from an outbreak in the Lower Hudson Valley, New York in 2016 (18), including NY230_16 (accession no. KY385890, positions 6862 to 6993), NY172_16, NY141_16, and NY135_16, and other genomes, including NY75_16 (85) and USA/TX/2016-19506, and USA/FL/2016-19504 (52). These latter two genomes were isolated from confirmed AFM cases (52). For the paired reads from 48145, the forward read aligns to the p2A protease gene for 35 bases and the p2B polypeptide gene for 58 bases (accession no. KY385890, positions 3665 to 3757 with two SNPs). The best BLAST hit is to three 2015 genomes from Osaka City, Japan (accession no. LC107898, LC107899, and LC107901) (86), with one SNP. The reverse read aligns to the p2B polypeptide gene (accession no. KY385890, positions 3799 to 3891 with one SNP). Best BLAST hits include many genomes, all with one SNP.

In addition to the metagenomic analysis pipelines, we performed a specific query for any reads matching EV-D68 in the metagenomic data with ASAP, utilizing bowtie2 (29) with no read clipping. EV-D68-specific reads were found in two samples, from patients 1 and 3, which were also EV-D68 positive by both real-time PCR and amplicon sequencing (Fig. 2). In sample 48136 (patient 1), one forward read and one reverse read (unpaired) aligned to the p3D RNA polymerase region. In sample 48145 (patient 3), a paired forward read and reverse read specifically matched EV-D68, aligning to p2A and p2B genes. BLAST analysis of these reads against the GenBank nucleotide database confirmed specificity of these reads to EV-D68.

## DISCUSSION

Although EV-D68 has not been recognized as a definitive cause of AFM at this time (14), multiple lines of evidence of EV-D68-associated AFM have been reported: direct viral detection in patients (7–13, 16, 19, 30–32), a recent demonstration of Koch’s postulate in a mouse model with EV-D68 (33), fulfillment of most of the Bradford Hill causation criteria by two independent analyses (34, 35), and most recently, the establishment that multiple EV-D68 strains from the 2014 outbreaks are neurotropic (i.e., infect and replicate in human neuronal cells) (36). Epidemiologically, EV-D68 has been shown to have a biennial circulation pattern in Europe (37) and in the United States, with increased incidence in 2014, 2016, and seemingly in 2018 (38) recapitulating the patterns of increased AFM reports in the same years (17). The suspected AFM cluster in Phoenix, AZ, in 2016 provided a unique opportunity to genomically explore AFM patient samples for the presence of EV-D68.

Our PCR and sequencing results found that NP swabs from all three confirmed AFM patients for which swabs were available were positive for EV-D68, and metagenomic data contained EV-D68 sequence in two of the three, adding to the strength and consistency of evidence supporting an EV-D68 etiology of AFM (34, 35). No NP swabs were available from the fourth confirmed case or single “probable” case. Not all published AFM cases show evidence of EV-D68, suggesting possible additional causes. The confirmed AFM patient for whom no NP swab was available was previously identified to be stool positive for coxsackievirus (20), a possible cause for AFM (35). However, EV-D68 may be present and yet go undetected in many cases, and we illustrate that different methods and analyses result in variable detection of EV-D68. In this case cluster, the clear detection of EV-D68 by real-time PCR required a preamplification step. Metagenomic analyses were performed mainly for the purpose of identifying potential alternative etiologies and resulted in detection of a limited presence of EV-D68 reads, suggesting that viral RNA was present at very low levels, possibly due to actual low viral loads or RNA degradation. (Further discussion of the metagenomic results can be found in the supplemental material.)

Previous studies have determined that CSF is not a highly useful specimen type for EV-D68 detection (39). Despite detection from NP swabs, we did not detect EV-D68 in the CSF specimens from the AFM patients, an outcome that has been frequently reported (7, 11, 13, 40). EV-D68 has been documented in CSF in limited cases, including (i) a fast-progressing, fatal case of meningomyeloencephalitis (41), (ii) an undescribed case of acute flaccid paralysis in 2005 (42), (iii) a hematopoietic stem cell transplant recipient on mild immunosuppressants (43), (iv) a single patient in an Argentinian cluster (30), and (v) a CSF specimen contaminated with blood cells (limiting conclusions as to the nature of the viral infection) (14). Other neuropathic enteroviruses are also rarely detected in CSF (39, 44), including poliovirus (https://www.cdc.gov/polio/us/lab-testing/diagnostic.html; 45); therefore, testing recommendations for suspect viral neurologic illness typically include collection of stool and/or NP swabs (46, 47). EV-D68 viremia is an exceedingly rare finding; a recently developed EV-D68 mouse model suggests viral dissemination occurs via a direct neural pathway rather than via a hematogenous route (33), although Greninger et al. did report EV-D68 in the blood of one child diagnosed with AFM (7).

Several hypotheses have been suggested to explain the neurologic pathology, given the low incidence of direct neurologic infection with EV-D68. AFM has been proposed to be caused by an aberrant immune response in some patients, elicited by the enteroviral infection (7); virus-induced autoimmune damage is a well-documented etiology of neurologic disease (47). However, it has been noted that clinical and neuroradiographic findings are more consistent with EV-D68 neuroinvasion (11, 14), and as demonstrated in a mouse model, there was no beneficial effect of immunosuppression with steroids, while immunotherapy containing anti-EV-D68 antibodies was effective, countering the autoimmune hypothesis (48). Another proposed hypothesis is that differences in viral infection outcomes are likely due to complex combinations of viral genotype, interaction of virus with respiratory mucosa, its microbiome, host immunity (modulated partly by the microbiome), and/or environmental factors (49). Recent clusters of AFM have been associated with widespread strains in particular phylogenetic clades. Genomes from the 2012 to 2014 EV-D68 spike in the United States formed a new clade, B1, nested within clade B and characterized by particular mutations that were hypothesized to elevate virulence (7, 36, 50), while 2014 genomes from other countries formed a related clade, B3, also nested within clade B with unique mutations (51). EV-D68 circulating in 2016 belonged to clade B3 (15, 18, 19), including isolates detected in sporadic cases of AFM (52). Hixon et al. used a mouse model to show four out of five 2014 EV-D68 isolates from clades A, B, and B1 caused paralysis using intracerebral injection, and a clade B1 isolate rarely caused paralysis using intranasal inoculation. Brown et al. recently demonstrated that strains from multiple clades displayed neuroinvasive capabilities in human neuronal cell lines (36). Greninger et al. documented EV-D68 infections in which one sibling suffered subsequent AFM, while the other did not (7). These observations suggest that neurologic outcomes are not necessarily strain driven. In another study, enteric viruses (e.g., poliovirus) in mice have been shown to exploit gut bacteria in order to facilitate host infection, and mice with depleted gut microbes were less susceptible (53). Disease severity of other viral infections (e.g., respiratory syncytial virus [RSV]) has been correlated with NP microbiome composition and host immune factors in children (54). However, our sample set did not include healthy controls and was too small to correlate microbiome structure with disease. Regardless of the factors involved, the likelihood that EV-D68 infection results in neurologic disease appears similar to that of other enteroviruses, such as poliovirus (4).

Our finding of the normally quiescent human endogenous retrovirus K (HERV-K) in the patient CSF samples (see the supplemental material) may add to the complexity of AFM and other neurologic manifestations. However, as our yield of RNA after DNase treatment and prior to RNA amplification was very low (data not shown), we suspect that residual DNA may be confounding these findings. Human genomic DNA harbors many HERV proviruses or remnants thereof (55); thus, we interpret our metagenomic hits to HERV-K with caution. Nevertheless, activation of HERVs by exogenous viruses (e.g., herpesviruses and HIV) is well documented (55, 56). Endogenous retroviruses have been shown to cause or exacerbate disease in some mammal species (55), and the HERV-K envelope protein is known to cause neuronal degeneration *in vitro* and *in vivo* (52). Additionally, antiretroviral therapy quelled HERV-K expression and amyotrophic lateral sclerosis (ALS)-like motor neuron disease in HIV patients (57). These observations ought to prompt further investigation in this area to potentially offer new insight into virus-induced neurologic disease. Notably, our observations also highlight one drawback to the removal of human data, which would include endogenous proviruses, from an RNA data set before analysis, as is common with metagenomic analysis tools.

The confirmed AFM patient (patient 4) who did not have evidence of EV-D68 (for which an NP swab was not available for analysis) did have HSV DNA in the CSF (sample 48127) by MTS bioinformatics analysis (see the supplemental material). The treating physician also noted the presence of a cold sore in this patient. The initial diagnostic screen for HSV in CSF was negative; however, false-negative HSV PCR results are often observed (47, 58). HSV is an established etiologic agent of other neurological diseases (47), is known to take a neural route of invasion of the central nervous system (CNS), and can reside at relatively high levels in the CSF of patients with HSV-caused encephalomyelitis (47). Discovery of HSV DNA in the metagenomic CSF data for this patient supports a possible HSV etiology in this case.

The single non-AFM EV-D68-positive sample (48139) was an NP swab from a patient whose differential diagnoses included ADEM or MS at the time of sample collection. Enteroviruses are a known cause of ADEM (59–63), including type D68 specifically (60). Additionally, ADEM and MS have overlapping diagnostic criteria with AFM. Among the many diagnostic criteria, ADEM is characterized by lesions in the white and gray matter evident from magnetic resonance imaging (MRI), and the lesion patterns are variable (64).

For this study, we took several highly sensitive molecular-based approaches: first to check for the presence of EV-D68 in a cluster of children suffering neurologic symptoms with suspect AFM and second to assess samples for other possible etiological causes. In the former approach, though the sample set was small, our sensitivity, specificity, and overall agreement measures show that the EV-D68 assay results are consistent with AFM diagnosis; however, there is confounding between AFM diagnosis and availability of NP swabs. A discordance rate between AFM in NP and CSF samples would be required to establish assay validation of EV-D68 and AFM diagnosis. In the latter approach, in addition to finding variability in detection of EV-D68 among the analysis methods (discussed above), we found variability among results generated by the 16S microbiomic and metagenomic analyses. The 16S sequence analysis was performed on DNA extracted from specimens, while most of the metagenomic data (save four samples) were generated from RNA extracted from specimens. Additionally, all of the metagenomic analyses we performed counted only species-specific sequence, with any reads that originated from any one of multiple species removed to prevent misleading results. The difference in the material recovered through each extraction method and the vast difference in the analysis of each approach likely explain much of the difference between results from 16S and metagenomic analyses. Among the metagenomic analyses, different databases and read filtering criteria were likely responsible for the relatively small differences in results for the same NP swab data sets. For the CSF samples, as the vast majority of data were host sequence, the relatively small amounts of contaminant microbial data were almost solely in the MTS results.

Identification of the etiology of AFM and related illnesses is important in order to understand risk factors, focus surveillance efforts, properly treat diagnosed AFM patients, and to help limit future outbreaks. Emphasis must be placed on the timely collection and appropriate handling of patient specimens in order to increase the likelihood of detection of RNA viruses—in this case EV-D68 (7, 9, 14, 30). The severity and outcome of AFM are devastating (3, 65), and delayed detection and improper management could worsen outcome (11). The use of multiple molecular and bioinformatic tools is still necessary until preferred sample types and definitive diagnostic markers are identified. Additionally, acknowledgment of EV-D68 as a likely etiologic agent of AFM could allow for improved surveillance and response and provide support for resource expenditure for vaccine development to eventually prevent AFM or other EV-D68 neurologic disease.

## MATERIALS AND METHODS

### Cluster identification and sample collection.

In August and September 2016, 11 children presented to a Maricopa County (Phoenix), AZ, facility with focal limb weakness, all but one having immediate prior respiratory, febrile, and/or gastrointestinal illness. Initial differential diagnoses included transverse myelitis and AFM (20). As part of standard of care, cerebrospinal fluid (CSF) samples were collected from all patients between 1 and 32 days from onset of focal limb weakness, and nasopharyngeal (NP) swabs were collected from 6 of the 11 patients 1 to 14 days following onset of illness. Samples were submitted for testing and then stored at −20°C. Sample and testing information is included in Tables 1 and 2. The CSF and NP swab samples were deidentified and coded for subsequent genomic analyses, and institutional review board approval was not required, as the remnant samples were used for public health surveillance. Chart reviews and patient interviews were conducted during the course of sample analysis; therefore, all genomic analyses were blind. AFM was clinically diagnosed according to the 2015 CDC case definition, as described previously (20). The leading differential diagnosis at the time data were abstracted was recorded for the non-AFM case children (Table 1).

### Nucleic acid extraction.

DNA was extracted from 200 to 400 μl of each patient sample with the DNeasy blood and tissue kit (Qiagen) using the Gram-positive protocol in the supplied handbook with some modifications. Initial lysis was extended to 60 min at 37°C, and secondary lysis was performed with proteinase K at 56°C for 30 min. RNA was extracted from 100 to 400 μl of specimen with the High Pure viral RNA kit (Roche).

### Real-time PCR and targeted amplicon sequencing.

EV-D68 VP-1 sequences from 2014 to 2016 were collected from NCBI’s nucleotide database. Sequences were aligned in SeqMan (DNAStar) to identify conserved regions for primer design, and assays were designed with guidance from RealTimeDesign (Biosearch Technologies). Each primer and probe was run through BLAST (http://blast.ncbi.nlm.nih.gov/Blast.cgi) to check for cross-reactivity to other relevant targets or species, including other enteroviruses and humans. The assay, listed in Table 3 and mapped in Fig. 2, results in a 94-bp amplicon. For amplicon sequencing, a universal tail sequence was added to each primer (Table 3) (20).

First-strand cDNA synthesis of the total RNA was performed with a high-capacity cDNA reverse transcription kit (Thermo Fisher Scientific). Preamplification, which has been shown to greatly increase sensitivity in complex samples (66, 67), was performed using the TaqMan PreAmp master mix (Thermo Fisher Scientific) with the EV-D68 primers (Table 3) at a final concentration of 5 to 10 nM. Real-time PCR was run on the 7900HT (Thermo Fisher Scientific) in 10-μl reaction mixtures containing 5 μl PerfeCTa FastMix II, 400 nM each forward primer, 600 nM each reverse primer, 200 nM each probe, and 4 μl preamplified template, with denaturation at 95°C for 3 min and 40 cycles of 95°C for 15 s and 60°C for 1 min.

Amplicon library preparation using universal tails was described previously (68). The initial gene-specific PCR comprised 12 μl 2× Kapa 2 G Fast Multiplex Mastermix (Kapa Biosystems), 10 μl primer mix yielding a final PCR concentration of 200 nM each, and 2 μl DNA template from each sample, and was denatured at 95°C for 3 min and then cycled 20 times at 95°C for 15 s, 60°C for 30 s, and 72°C for 1 min 30 s, with final extension at 72°C for 1 min. A second PCR using the universal tail-specific primers (Table 3) added Illumina’s sample-specific index and sequencing adapters. This PCR comprised 12.5 μl 2× Kapa HiFi HotStart ReadyMix (Kapa Biosystems), universally tailed forward and reverse primers at 400 nM each, and 10.5 μl cleaned gene-specific PCR product for a final volume of 25 μl, and was denatured at 98°C for 2 min and then cycled 8 times for RNA metagenomic samples and 12 times for DNA at 98°C for 30 s, 65°C for 20 s, and 72°C for 30 s, with final extension 72°C for 5 min. Final PCR products were cleaned with 1× Agencourt AMPure XP beads (Beckman Coulter). Amplicon libraries from individual samples were quantified by quantitative PCR (qPCR) using the Kapa library quantification kit (Kapa Biosystems) and then pooled in equimolar concentration for sequencing on the Illumina MiSeq platform with the 2× 250-bp version 2 kit.

### 16S microbiome library preparation, sequencing, and analysis.

Partial 16S rRNA genes in each metagenomic sample were amplified by PCR and prepared for sequencing as described previously (69) using the primer pair S-d-Bact-0341-b-S-17 and S-d-Bact-0785-a-A-21 (70) with universal tail sequences (Table 3), resulting in an amplicon of 481 bp that spans the V3 and V4 regions. Amplification was performed in a 25-μl reaction volume containing 12.5 μl Q5 Hot Start high-fidelity 2× master mix (New England Biolabs, Inc.), 500 nM each primer, and 5 to 10 μl of DNA using thermal conditions previously described (69). Amplicons were purified using the Agencourt AMPure XP beads (Beckman Coulter) following the manufacturer’s protocol. To separate the bacterial 16S amplicon from the human mitochondrial amplicon (69), the samples were processed through the BluePippin DNA size selection system (Sage Science). The index PCR comprised 12.5 μl of Kapa HiFi HotStart ReadyMix (Kapa Biosystems), 400 nM each indexing primer specific to each universal tail, and 10 μl of DNA in a final reaction volume of 25 μl run at 98°C for 2 min and then 10 cycles at 98°C for 30 s, 65°C for 20 s, and 72°C for 30 s, with a final hold at 72°C for 5 min. Indexed libraries were purified, quantified, and pooled as described above, and sequenced on the Illumina MiSeq platform with the 2× 300-bp version 3 kit.

Bacterial community content and diversity were examined with QIIME (71), using uclust (72) to pick operational taxonomic units (OTUs), PyNAST (73) to align reads to the Greengenes 16S gene database version 13_8 (74), ChimeraSlayer (75) to detect and filter chimera sequences, and the Greengenes taxonomic classification system (76) to assign taxonomy. A DNA extraction blank and a 16S PCR reagent blank were included in the sample preparation and analysis.

### DNA and RNA metagenomic library preparation, sequencing, and analysis.

Total RNA was subjected to DNase I treatment and concentration using RNA Clean and Concentrator-5 (Zymo Research) and then amplified using the SeqPlex RNA amplification kit (Sigma-Aldrich). Total DNA was subject to fragmentation using a Q800R2 sonicator (QSonica). RNA and DNA metagenomic sequence libraries were prepared for sequencing and quantified using the Kapa Hyper Prep kit and Kapa library quantification kit (Kapa Biosystems). RNA and DNA libraries were sequenced on the Illumina HiSeq 2500 at 2× 100 bp using v3 chemistry. To deposit nonidentifying data in NCBI’s SRA database, computational subtraction of human sequence data was performed. For this, raw reads were aligned to a human genome (taxID 9606) using bowtie (29) version 2.2.2 with default parameters, and SAMtools (77) version 1.4.1 was used to extract only paired reads where neither read mapped to the human genome. Finally, BEDTools (78) version 2.62 was used to reconstitute the extracted reads into FASTQ format. These data were submitted to the SRA database. By these methods, viral sequence data that may be present in the human genome reference sequence (e.g., herpesviruses and HERVs) are subtracted along with human sequence data.

Three metagenomic analysis tools, MTS (A. Perry, T. Schneider, and V. Fofanov, poster, presented on 21 June 2017 at the Qiime2 Workshop [https://workshops.qiime2.org/] in Las Vegas, NV), GOTTCHA (79), and MetaPhlAn (80), were employed for thorough taxonomic classification of reads from each sample; the details of which can be found in the supplemental material. These three methods represent the spectrum of potential databases that can be used for metagenomic data query, ranging from the most inclusive (MTS, which uses the entire NCBI GenBank microbial database) to the most focused (MetaPhlAn, which uses marker genes only). For all metagenomic analyses, background results from a blank were subtracted from the sample data where appropriate.

### EV-D68 amplicon and metagenomic data targeted analysis.

Amplicon and metagenomic sequencing results were analyzed using the automated amplicon sequencing analysis pipeline ASAP, as described in detail previously (68, 81, 82). Briefly, amplicon or metagenomic sequence reads were first trimmed of adapter and read-through sequences with Trimmomatic (83) and then mapped to a reference sequence with bowtie2 (29). Amplicon sequence was mapped to the PCR amplicon region of a 2016 EV-D68 VP1 gene (GenBank accession no. KY385890). Metagenomic data were mapped to the EV-D68 whole-genome sequence (accession no. KY385890). Tablet (84) was used to verify results.

### Data availability.

EV-D68 assay amplicon data have been deposited in NCBI’s SRA database under BioProject no. PRJNA377726 (20). Amplicon read data and metagenomic data were deposited in NCBI’s SRA under BioProject no. PRJNA474932. Viral sequence data will be made available upon request.
