# Supplementary material for: Genomic Analyses of Acute Flaccid Myelitis Cases among a Cluster in Arizona Provide Further Evidence of Enterovirus D68 Role
Source: mBio. 2019 Jan 22;10(1):e02262-18. doi: 10.1128/mBio.02262-18 (PMC6343034; doi:10.1128/mBio.02262-18)
Supplement: TEXT S1 [file mBio.02262-18-s0001.docx]

**Supplemental Material**

**Methods**

*Metagenomic Bioinformatic Analysis*

Three metagenomic analysis tools, MTS (1), GOTTCHA (2), and MetaPhlAn (3) were employed for thorough taxonomic classification of reads from each sample to take multiple approaches to search for possible disease etiologies, and in order to address the major challenges that arise during taxonomic classification of short high throughput sequencing (HTS) reads, e.g. the significant rates of taxonomic misclassification that arise due to the inevitable genomic similarities among organisms (4-6) and the inaccuracies in publicly available genomic reference databases, which have been shown to contain taxonomically misclassified or contaminant sequences (7-9). MTS ([**https://github.com/FofanovLab/MTSv**](https://github.com/FofanovLab/MTSv)) (1) was designed to address the extreme computational expense of sequence read alignment to reference databases and false positive potential. MTS uses a local copy of NCBI’s GenBank database generated by downloading the flat files of the entire NCBI GenBank, and discarding all sequences without a valid (species level or below) taxonomic classification or those labeled as “environmental” and all metadata other than GI/Accession and TaxID. The resulting sequences are built into a custom FM-Index for fast taxonomic classification. In MTS, most human sequence data were computationally subtracted allowing two mismatches per read. The remaining paired-end reads were decoupled to ignore pairing information, segmented into non-overlapping 45-mers, and aligned to the MTS database (built from GenBank 02/09/2017), using bowtie’s alignment algorithm (10), which aligns reads while eschewing identification of where and how many times a given read aligns within a given species’ genome, providing for a significantly faster first hit query within all reference sequences of a given species. The resulting alignment speedup (up to 1,000X) using MTS allows identification of all species to which any given read aligns. Only reads that unambiguously align to one and only one species were counted in the taxonomic composition of a sample, significantly reducing false positive hits. Lastly, MTS post-filtering of alignments was carried out using a modified MinHash (MASH) (11) procedure, using short n-mers to generate pairwise distances between all reference sequences in a taxonomic unit, thereby identifying sequences potentially misclassified in Genbank. Secondly, GOTTCHA (<http://lanl-bioinformatics.github.io/GOTTCHA/>) (2) minimizes false positive hits and gives accurate taxonomic abundances by providing “unique” pre-computed viral, bacterial, and human databases at species, genus, and family levels. Briefly, reference genomes from a curated version of NCBI’s Genbank are pooled at species/genus/family/etc. level, disassembled into all possible 30-mers, and used to compute a no-mismatch intersection between every species/genus/family/etc. Only the 30-mer fragments that are present in only one species (or genus/family, depending on the level of the database) are kept for further analysis. The prokaryotic and viral database versions used in GOTTCHA were v20120316 and v20141222 respectively, and default parameters, using BWA (12) (version 0.7.12-r1044 in this study), were used for analysis. Lastly, MetaPhlAn2 (<https://bitbucket.org/biobakery/metaphlan2>) (3) preselects a significantly reduced marker library that is representative of a given clade or operational taxonomic unit (~1M unique clade-specific marker genes) from sequences identified from ~17,000 reference genomes (~13,500 bacterial and ~3500 viral). This reduced reference database obviates pre-processing of sample sequence data, achieving rapid classification, generating only high confidence hits (at the cost of potential loss of sensitivity). For MetaPhlAn analysis, default parameters were selected.

**Results**

*Metagenomic Bioinformatic Analysis*

Three different sequence analysis tools (i.e., MTS, GOTTCHA, and MetaPhlAn) were employed to characterize the organism composition in each metagenomic sample for robustness and comprehensiveness. MTS analyses in general identified more microbes than the other metagenomic analyses. *Micrococcus luteus* was the top non-human hit in all CSF RNA samples and the control and *Acinetobacter guillouiae* was consistently identified among the top five hits with MTS (Table S1), both common laboratory contaminants (13). These species were absent or at very low levels in the 16S analysis, and were not in the DNA metagenomic analyses, indicating likely contamination during the total RNA sample preparation. Other signature hits included the skin microflora *Propionibacterium acnes* and *Staphylococcus epidermidis*, also noted in the 16S microbiomic analysis. Nearly all top hits found in the NP swab metagenomic data matched those found in the 16S data. One sample, 48136, yielded no signature hits. HSV RNA was not identified in any RNA sample.

GOTTCHA analysis largely agreed with the MTS results for the NP swabs, although in some cases (48137 and 48132 from Patients 2 and 7) MTS identified more species, and in other cases (48145 and 48147 from Patients 3 and 11) GOTTCHA identified an organism in the DNA that MTS detected only in the RNA (Table S1). GOTTCHA additionally identified in all CSF RNA samples and several NP swabs human endogenous retrovirus K (HERV-K), which has been implicated in neurodegeneration (14), and its expression has been associated with amyotrophic lateral sclerosis (ALS)(14, 15), MS (16, 17), rheumatoid arthritis (RA) (17), schizophrenia, HIV-associated dementia, and cancer (18). One CSF RNA sample (48138, from AFM Patient 2 whose NP swab was EV-D68 positive), and one NP RNA sample (48136, also EV-D68 positive from AFM Patient 1) were each positive for human herpesvirus- (HHV-) 6A.

MetaPhlAn results included several additional bacterial identifications in the NP swabs not found with MTS or GOTTCHA, though many species were of the same genera (Table S1). MetaPhlAn, like GOTTCHA, identified HERV-K in all CSF samples and several NP swabs. MetaPhlAn did not identify HSV or HHV in any CSF sample.

**Discussion**

*Metagenomic Analysis*

It is possible we did not detect the VP1 region of the EV-D68 genome (detected by the PCR) in the metagenomic data for various reasons, including: i) as short DNA or cDNA fragments (<150 bases) were selectively removed when preparing samples for metagenomic sequencing, the EV-D68 VP1 fragments may have been removed during the fragment size-selection part of the sample preparation process, or ii) given the overwhelming amount of human genomic material in the sample, the depth of unbiased sequencing may not have been large enough to have included EV-D68 sequence. Under assumptions of random sampling and a 1:1 mixture of EV-D68 to human host genome copies, the probability of observing at least 100 EV-D68 reads from a 17.5 million-read pair run (in four of our samples) is essentially 0%. However, for bacteria with a genome size of ~2.5 Mbp, even at our lowest sequencing depth of 0.32 million read pairs, the probability of obtaining at least 100 reads from the target genome is 100%. In fact, at this sequencing depth we have 76.2% chance of obtaining >250 reads from the bacterial target, which is well within the detection threshold for MTS and GOTTCHA taxonomic classification approaches. Indeed, metagenomic analyses in this study were performed mainly for the purpose of identifying potential alternative etiologies.

Besides detection of HSV in Patient 4 (who also has a noticeable cold sore), low-level HSV sequence reads were also found by MTS in the CSF of a patient diagnosed with NMO and the NP swab of an AFM patient (Patient 1). A different analysis tool (GOTTCHA) also identified herpesvirus in the RNA of Patient 1’s NP swab (150 reads) and in the RNA of CSF from Patient 2 (128 reads), but labeled it as HHV-6A, a potentially neuroinvasive herpesvirus (19). Although not historically found in the U.S., HHV-6A symptomatic infection has recently been reported in children from the U.S. (20). It is plausible the herpesviruses directly caused or exacerbated the neurologic pathologies in these cases, that the viruses were activated by other causes of the neurologic disease (i.e, coxsackievirus in Patient 4), or that we detected inactive HHV DNA in the hosts’ genomes.

References:

1. **Perry A, Schneider T, Fofanov V.** 2017. MetaSeQ: Fast Metagenomic Binning. Poster presentation. June 21, 2017, Las Vegas, NV.

2. **Freitas TA, Li PE, Scholz MB, Chain PS.** 2015. Accurate read-based metagenome characterization using a hierarchical suite of unique signatures. Nucleic Acids Res **43:**e69.

3. **Segata N, Waldron L, Ballarini A, Narasimhan V, Jousson O, Huttenhower C.** 2012. Metagenomic microbial community profiling using unique clade-specific marker genes. Nat Methods **9:**811-814.

4. **Ackelsberg J, Rakeman J, Hughes S, Petersen J, Mead P, Schriefer M, Kingry L, Hoffmaster A, Gee JE.** 2015. Lack of Evidence for Plague or Anthrax on the New York City Subway. Cell Syst **1:**4-5.

5. **Afshinnekoo E, Meydan C, Chowdhury S, Jaroudi D, Boyer C, Bernstein N, Maritz JM, Reeves D, Gandara J, Chhangawala S, Ahsanuddin S, Simmons A, Nessel T, Sundaresh B, Pereira E, Jorgensen E, Kolokotronis SO, Kirchberger N, Garcia I, Gandara D, Dhanraj S, Nawrin T, Saletore Y, Alexander N, Vijay P, Henaff EM, Zumbo P, Walsh M, O'Mullan GD, Tighe S, Dudley JT, Dunaif A, Ennis S, O'Halloran E, Magalhaes TR, Boone B, Jones AL, Muth TR, Paolantonio KS, Alter E, Schadt EE, Garbarino J, Prill RJ, Carlton JM, Levy S, Mason CE.** 2015. Modern Methods for Delineating Metagenomic Complexity. Cell Syst **1:**6-7.

6. **Afshinnekoo E, Meydan C, Chowdhury S, Jaroudi D, Boyer C, Bernstein N, Maritz JM, Reeves D, Gandara J, Chhangawala S, Ahsanuddin S, Simmons A, Nessel T, Sundaresh B, Pereira E, Jorgensen E, Kolokotronis SO, Kirchberger N, Garcia I, Gandara D, Dhanraj S, Nawrin T, Saletore Y, Alexander N, Vijay P, Henaff EM, Zumbo P, Walsh M, O'Mullan GD, Tighe S, Dudley JT, Dunaif A, Ennis S, O'Halloran E, Magalhaes TR, Boone B, Jones AL, Muth TR, Paolantonio KS, Alter E, Schadt EE, Garbarino J, Prill RJ, Carlton JM, Levy S, Mason CE.** 2015. Geospatial Resolution of Human and Bacterial Diversity with City-Scale Metagenomics. Cell Syst **1:**97-97 e93.

7. **Merchant S, Wood DE, Salzberg SL.** 2014. Unexpected cross-species contamination in genome sequencing projects. PeerJ **2:**e675.

8. **Schmieder R, Edwards R.** 2011. Fast identification and removal of sequence contamination from genomic and metagenomic datasets. PLoS One **6:**e17288.

9. **Tao ZY, Sui X, Jun C, Culleton R, Fang Q, Xia H, Gao Q.** 2015. Vector sequence contamination of the Plasmodium vivax sequence database in PlasmoDB and In silico correction of 26 parasite sequences. Parasit Vectors **8:**318.

10. **Langmead B, Salzberg SL.** 2012. Fast gapped-read alignment with Bowtie 2. Nat Methods **9:**357-359.

11. **Ondov BD, Treangen TJ, Melsted P, Mallonee AB, Bergman NH, Koren S, Phillippy AM.** 2016. Mash: fast genome and metagenome distance estimation using MinHash. Genome Biol **17:**132.

12. **Li H, Durbin R.** 2009. Fast and accurate short read alignment with Burrows-Wheeler transform. Bioinformatics **25:**1754-1760.

13. **Salter SJ, Cox MJ, Turek EM, Calus ST, Cookson WO, Moffatt MF, Turner P, Parkhill J, Loman NJ, Walker AW.** 2014. Reagent and laboratory contamination can critically impact sequence-based microbiome analyses. BMC Biol **12:**87.

14. **Li W, Lee MH, Henderson L, Tyagi R, Bachani M, Steiner J, Campanac E, Hoffman DA, von Geldern G, Johnson K, Maric D, Morris HD, Lentz M, Pak K, Mammen A, Ostrow L, Rothstein J, Nath A.** 2015. Human endogenous retrovirus-K contributes to motor neuron disease. Sci Transl Med **7:**307ra153.

15. **Bowen LN, Tyagi R, Li W, Alfahad T, Smith B, Wright M, Singer EJ, Nath A.** 2016. HIV-associated motor neuron disease: HERV-K activation and response to antiretroviral therapy. Neurology **87:**1756-1762.

16. **Morandi E, Tanasescu R, Tarlinton RE, Constantinescu CS, Zhang W, Tench C, Gran B.** 2017. The association between human endogenous retroviruses and multiple sclerosis: A systematic review and meta-analysis. PLoS One **12:**e0172415.

17. **Hohn O, Hanke K, Bannert N.** 2013. HERV-K(HML-2), the Best Preserved Family of HERVs: Endogenization, Expression, and Implications in Health and Disease. Front Oncol **3:**246.

18. **Christensen T.** 2016. Human endogenous retroviruses in neurologic disease. APMIS **124:**116-126.

19. **De Bolle L, Van Loon J, De Clercq E, Naesens L.** 2005. Quantitative analysis of human herpesvirus 6 cell tropism. J Med Virol **75:**76-85.

20. **Ablashi D, Agut H, Alvarez-Lafuente R, Clark DA, Dewhurst S, DiLuca D, Flamand L, Frenkel N, Gallo R, Gompels UA, Hollsberg P, Jacobson S, Luppi M, Lusso P, Malnati M, Medveczky P, Mori Y, Pellett PE, Pritchett JC, Yamanishi K, Yoshikawa T.** 2014. Classification of HHV-6A and HHV-6B as distinct viruses. Arch Virol **159:**863-870.
